# RIP3‐mediated necroptosis increases neuropathic pain via microglia activation: necrostatin‐1 has therapeutic potential

**DOI:** 10.1002/2211-5463.13258

**Published:** 2021-08-24

**Authors:** Ping Fang, Gangqiang Sun, Jingyu Wang

**Affiliations:** ^1^ Department of Anesthesiology Ningbo Medical Treatment Center Lihuili Hospital China

**Keywords:** microglia, necroptosis, necrostatin‐1, neuropathic pain, receptor‐interacting protein kinase 3

## Abstract

Neuropathic pain (NP) is a clinical symptom that accompanies many diseases. We investigated the effect of receptor‐interacting protein kinase 3 (RIP3)‐regulated necroptosis on NP and explored its relationship with microglia, in order to provide a theoretical basis for further research and provide new insights into the treatment of NP. In this study, the spared nerve injury (SNI) model was used along with intervention with necrostatin and the inhibitor of necroptosis necrostatin‐1 (Nec‐1). Pain behavior tests were performed 1 and 3 days before the nerve injury (or sham) operation, and on days 1, 3, 5, 7, 10, and 14 after the operation. The spinal cord tissues were collected for detection of RIP3 expression and distribution, changes in the number of microglia cells, activation of necroptosis, and the level of proinflammatory factors. Collected spinal cord tissues were analyzed using western blot, immunohistochemistry, immunofluorescence, immunoprecipitation assays, and ELISA, respectively. We found that, compared with the sham group, the expression of RIP3 protein in the spinal cord of rats in the SNI group increased from 3 to 14 days after surgery. Immunofluorescence staining showed that RIP3 was coexpressed with the microglia and the number of microglia increased significantly in the SNI model group. The results of immunoprecipitation assays suggested that a RIP3‐mediated necroptosis pathway promotes NP. After treatment with Nec‐1, the expression of RIP3 protein and the number of microglia were significantly reduced, and the expression levels of TNF‐α, IL‐1β, and IL‐6 in spinal dorsal horns were significantly decreased. These results indicate that RIP3 promotes necroptosis to increase the occurrence of NP via microglia.

## Graphical abstract

Here, we report that receptor‐interacting protein kinase 3 (RIP3) is upregulated in a nerve injury (SNI) rat model, and the RIP3‐mediated necroptosis pathway promotes neuropathic pain (NP) in rats via microglia. Additionally, intrathecal injection of necrostatin‐1 reduces NP in SNI rats via regulation of microglia in the spinal cord and downregulation of proinflammatory factors. These results provide a theoretical basis for further research and provide new insights into the treatment of NP.

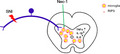

**Abbreviations**
IHC: immunohistochemistry
IL‐1β: interleukin‐1β
IL‐6: interleukin‐6
MLKL: mixed lineage kinase domain‐like protein
NP: neuropathic pain
PWMT: paw withdrawal mechanical threshold
RIP1: receptor‐interacting protein kinase‐1
RIP3: receptor‐interacting protein kinase 3
SNI: spared nerve injury
TBST: Tris‐buffered saline and Tween 20
TNF‐α: tumor necrosis factor‐α

Neuropathic pain (NP) refers to pain caused by damage or disease to the somatic sensory system. Studies have shown that its clinical prevalence can be as high as 10% and that it severely affects the quality of life of patients [1]. The molecular biological mechanism of NP is complex and the pathogenesis is still unclear, but the current research indicates that proinflammatory and inflammatory factors, cell apoptosis, receptors, ion channel changes, peripheral sensitization, and central sensitization are all involved in the generation and maintenance of NP [2, 3, 4, 5]. Among these factors, apoptosis and inflammation of spinal cord cells play especially important roles in the generation and maintenance of NP.

Under pathophysiological conditions, apoptosis and necroptosis are two different forms of cell death, but they are closely related to each other [6]. Necroptosis is considered to be a modifiable noncysteine aspartase‐dependent programmed cell death that can directly or indirectly cause inflammatory responses and plays an important role in the evolution of trauma, ischemia/reperfusion injury, neurological diseases, and infection [7, 8, 9, 10], but there is not much research on necroptosis in NP except reported by Liang *et al*. [11].

Here, in order to explore the role of necroptosis in NP, we studied the expression and role of the receptor‐interacting protein kinase 3 (RIP3), a key molecular switch connecting apoptosis, necrosis, and necroptosis [12], in the rat spared nerve injury (SNI) model, and observed the distribution of RIP3 protein in the spinal cord. At the same time, we monitored the effects of intrathecal administration of necrostatin‐1 (Nec‐1), an inhibitor of necroptosis, on pain behavior and inflammatory factors in rats. Our results provide a theoretical basis for further research and provide new insights into the treatment of NP.

## Materials and methods

### Animals and SNI model

Sprague Dawley male rats (200–250 g, 6 weeks old) were purchased from SLAC Laboratory Animal Co., Ltd (Shanghai, China). Animals were housed in a temperature‐controlled room (constant temperature of 21 ± 2 °C and a humidity of 60 ± 10%) with a 12‐h light/12‐h dark rhythm cycle and had free access to food and water. Surgery of the SNI model was performed according to the method outlined by Decosterd and Woolf [13]. Rats were anesthetized for surgery by intraperitoneal injection of 10% chloral hydrate 300 mg·kg^−1^. After the rats were fixed on the operating table, the upper edge of the left hind limb was disinfected and cut open, and. the muscle was separated bluntly to expose the main sciatic nerve and its branches. The common peroneal nerve and tibial nerve were ligated and incised with 5‐0 silk thread, leaving the small sural nerve intact, and finally, the muscle and skin were suturally closed. Technicians operated carefully in order to avoid pulling and damaging the sural nerve. After the operation, penicillin was injected intramuscularly to prevent infection. This research study design was approved by the Ethics Committee of Ningbo Medical Treatment Center Lihuili Hospital.

### Groups, drug administration, and pain behavior tests

To examine whether the RIP3 enrichment was altered on spinal dorsal horn tissues after SNI, 24 SD rats were randomly divided into two groups: a sham operation group (Sham group), and a model group (SNI group). Four rats of SNI group were euthanized at the third and the seventh day after the operation, respectively, the remaining rats were euthanized at the 14th day. To evaluate the effects of Nec‐1 on NP in SNI model and explore its underlying mechanism *in vivo*, 36 SD rats were divided into three groups: a sham operation group (Sham group) + DMSO, a model group (SNI group) + DMSO, and a model + administration group (Nec‐1 group), each with 12 rats. All rats underwent intrathecal catheterization in advance. One week after administration of the intrathecal tube, the surgical model was performed. Rats in the SNI group and the Nec‐1 group underwent SNI surgery to prepare SNI models, as described above. The Nec‐1 group was intrathecally injected with 5 μg Nec‐1 (Sigma, St. Louis, MO, USA) dissolved in 10 μL DMSO on days 5–10 after the operation, the Sham group and SNI group were intrathecally injected with 10 μL DMSO. After injection, the intrathecal tubes were flushed with 10 μL normal saline. Pain behavior tests were performed 1 and 3 days before the operation, and on days 1, 3, 5, 7, 10, and 14 after the operation, using the methods outlined by Chaplan *et al*. [14] to determine the paw withdrawal mechanical threshold (PWMT). Fourteen days after the operation, the rats were euthanized and L4‐L6 spinal dorsal horns were removed for molecular biological index detection.

### Immunoprecipitation and immunoblotting

The microglia cells were extracted from the spinal cords of rats by flow cytometry sorting after anti‐CD11b (Abcam, Cambridge, UK) staining and the cell samples were diluted to the same concentration with Radioimmunoprecipitation assay lysis buffer and extraction buffer. Then, the samples were precleared by incubation with protein G beads (20 μL; Amer‐sham Biosciences, Foster, CA, USA) for 2 h, followed by incubation with 1–2 μg of primary antibody RIP3 overnight at 4 °C. Protein G beads were then added to the tube for further incubation for 2 h. Samples were then centrifuged at 10 000 ***g*** for 1 min at 4 °C and the pellets were washed three times with immunoprecipitation buffer. For immunoblotting, bound proteins were eluted by boiling at 100 °C for 10 min in SDS/PAGE loading buffer and then isolated by centrifugation. The proteins were subjected to 12% SDS/PAGE electrophoresis and then transferred to a PVDF membrane. The membrane was blocked with 5% skim milk at room temperature for 2 h and then washed with Tris‐buffered saline and Tween 20 (TBST). The membrane was then incubated at 4 °C overnight with either anti‐RIP3 (CST, Danvers, MA, USA; 95702S), anti‐RIP1 (Abcam; ab72139), anti‐MLKL (Abcam; ab184718), anti‐β‐actin (CST; 4970S) primary antibodies. The primary antibodies were diluted to 1 : 500 in TBST. After washing, the membranes were subsequently incubated with secondary antibody for 1 h at 37 °C. An efficient chemiluminescence kit was used to visualize the protein bands. β‐actin was used as the loading control, as described in publications [15].

### Immunohistochemistry staining

RIP3 antibodies (Abcam; ab62344), were used at a dilution of 1 : 200 for the immunohistochemistry (IHC) detection of protein expression in the spinal cord from the enlarged part of L4‐6. Nonspecific staining was blocked with 0.5% casein and 5% normal serum. The samples were incubated with biotinylated antibodies and horseradish peroxidase. Staining was developed with diaminobenzidine substrate and the sections were counterstained with hematoxylin. Negative controls were obtained by using PBS instead of RIP3 antibody.

### Immunofluorescence

RIP3 (Abcam; ab62344), GFAP (Abcam; ab4648), and Iba‐1 (Santa Cruz, Dallas, TX, USA; sc‐32725) antibodies were used to detect RIP3, astrocyte cells, and microglia cells, respectively. Prior to immunofluorescence analysis, the sections were blocked with donkey serum albumin (Abbkine, Wuhan, Hubei, China; BMS0140) in PBS for 1 h at room temperature and then incubated at 4 °C with the primary antibodies. Then, the sections were incubated with secondary antibodies at 37 °C for 1 h. Slides were then mounted with Vectashield containing 4′,6‐diamidino‐2‐phenylindole and were visualized and imaged using a fluorescence microscope (Olympus Corporation, Tokyo, Japan) coupled to a CCD camera (Nikon Corporation). Negative controls, in which PBS was used in place of primary antibodies, were included for each marker.

### Enzyme‐linked immunosorbent assay

Fourteen days after SNI surgery, the samples were collected from the lumbar spinal cord to determine the levels of TNF‐α (Beyotime Biotechnology, Shanghai, China; PT516), IL‐1β (Beyotime Biotechnology, Shanghai, China; PI303), and IL‐6 (Beyotime Biotechnology, Shanghai, China; PI328). The specific experimental operation was carried out according to the instructions.

### Statistical analysis

Statistical analyses were performed in graphpad prism 8 software (La Jolla, CA, USA). The data were presented as mean ± standard deviation (SD). Student's *t*‐test and one‐way ANOVA were used for statistical analysis. The significance threshold for all of the experiments was set at *P* < 0.05.

## Results

### RIP3, the key regulator of necroptosis, is upregulated in the spared nerve injury rat model

**Fig. 1:**
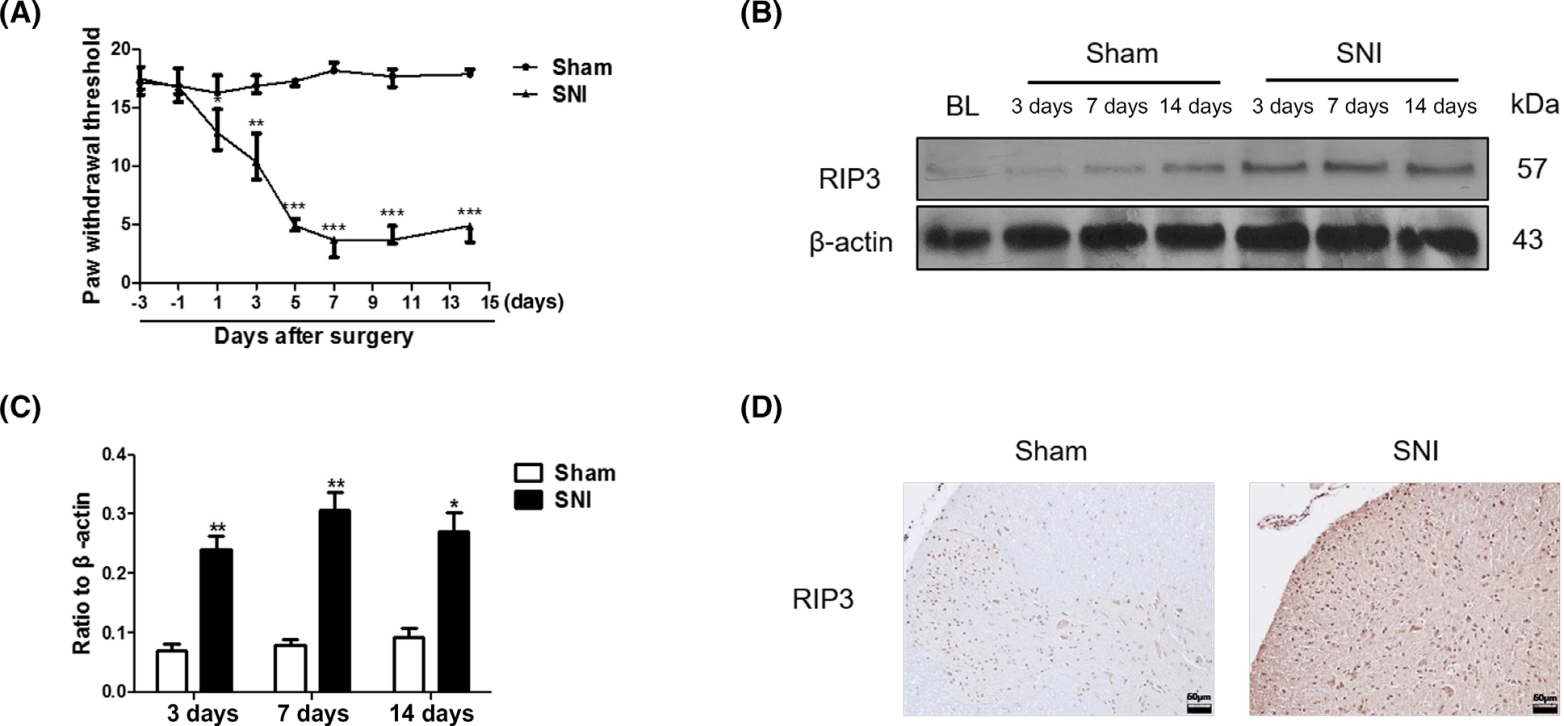
RIP3, the key regulator of necroptosis, is upregulated in the nerve injury (SNI) rat model. (A) PWMT analysis of the pain behavior in nerve injury rats (SNI group) and sham operation group (sham group). (B) Western blot analysis of the expression of RIP3 in the SNI rat model and Sham rat groups, 3, 7, and 14 days after operation on spinal dorsal horn tissue. (C) Ratio of RIP3 protein/β‐actin (the ratios were used to analyze significant difference). (D) RIP3 immunohistochemical staining in L4‐L6 spinal dorsal horns obtained from rats in the SNI and Sham groups 14 days after surgery (magnification, 200×; Scale bar: 50 μm), mean ± SD, *n* = 4 rats per group, *P < 0.05, **P < 0.01, ***P < 0.001 vs. Sham. Student's *t*‐test was used to assess the difference between two groups. All data were obtained from at least three independent experiments.

Changes in pain behavior were compared for rats in the sham group and the SNI model group from 3 days before operation to 14 days after operation. Pain behavior test results showed that there was no statistically significant difference in PWMT between the sham group and the SNI group 3 days before modeling and 1 day after the operation (*P* > 0.05); and 3 days after the operation, compared with the sham group, the PWMT of rats in the SNI group showed a gradual decrease, and the pain threshold fell to its lowest on the 7th day after surgery, and lasted until the 14th day after surgery (*P* < 0.001), and indicating that the SNI model was successfully established (Fig. 1A).

In order to verify the expression of RIP3 protein in SNI rats, spinal dorsal horn tissue was taken 3, 7, and 14 days after the operation, and western blot was used to detect the expression of RIP3 protein. The results showed that the expression of RIP3 protein in the spinal cord of rats in the sham operation group did not increase at any time points after operation (*P* > 0.05). Compared with the sham group, the expression of RIP3 protein in the spinal cord of rats in the SNI group increased at 3 days (*P* < 0.01) after surgery and was still elevated 14 days after surgery (*P* < 0.001) (Fig. 1B,C). Similarly, the IHC results showed that the expression of RIP3 protein in the spinal cord of rats in the SNI group was significantly higher than the expression in the sham group on the 14th day after surgery (Fig. 1D). These results indicate that the RIP3 protein is involved in the formation and development of NP.

### The RIP3‐mediated necroptosis pathway promotes neuropathic pain in rats via microglia

**Fig. 2:**
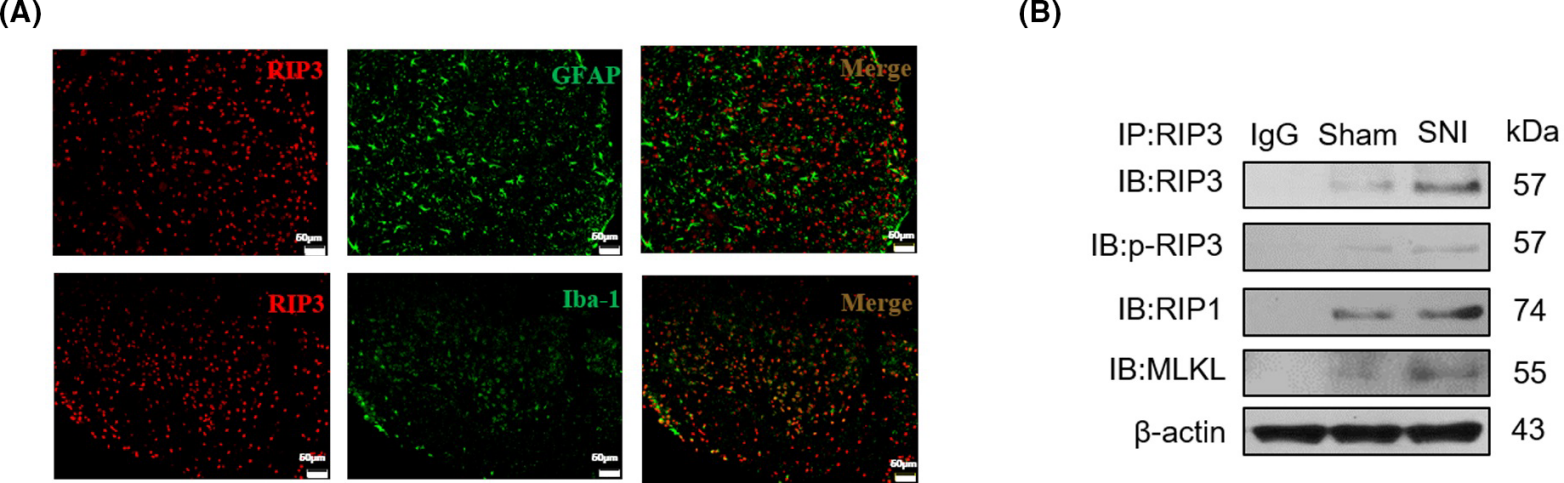
The RIP3‐mediated necroptosis pathway promotes NP in rats by activating microglia. (A) RIP3 protein was coexpressed with the microglia marker Iba‐1, but not with the astrocyte marker GFAP in the spinal cord of rats 14 days after surgery (magnification, 400×; Scale bar: 50 μm), *n* = 4 rats per group. (B) Interaction of RIP3 with RIP1, expression of RIP1, RIP3, and MLKL, and phosphorylation of RIP3 were measured by immunoblotting and immunoprecipitation using microglia cells from the spinal cords of rats sorted by flow cytometry. All data were obtained from at least three independent experiments.

**Fig. 3:**
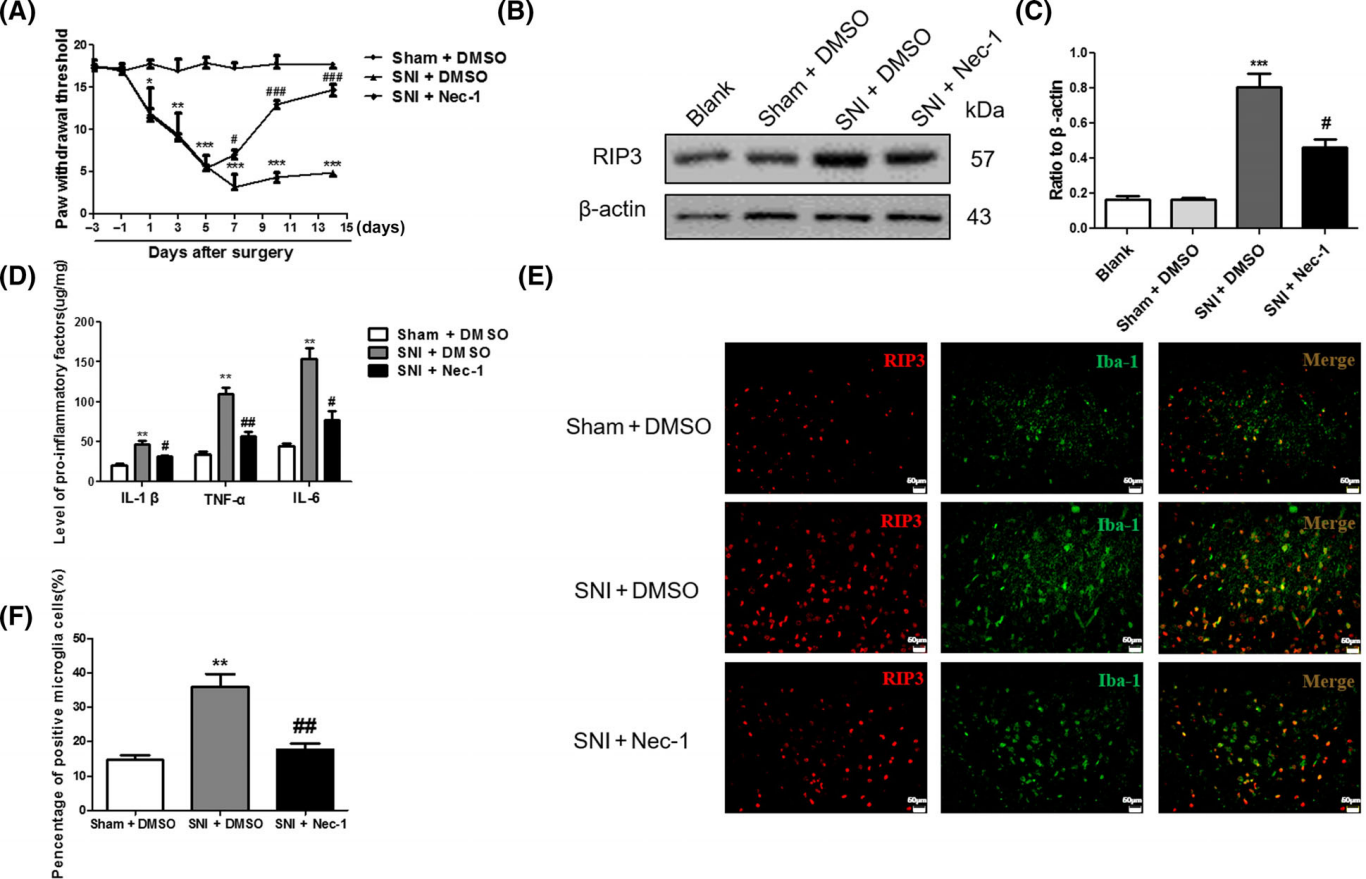
Intrathecal injection of the necroptosis pathway inhibitor Nec‐1 reduces NP in SNI rats. (A) PWMT analysis of pain behavior of rats in the sham group, SNI group, and Nec‐1 group. (B) Western blotting was used to detect the effect of Nec‐1 on the expression of RIP3 protein in SNI model rats in the spinal dorsal horn. (C) Ratio of RIP3 protein/β‐actin (the ratios were used to analyze significant difference). (D) ELISA analysis tests were used to detect the expression levels of TNF‐α, IL‐1β, and IL‐6 in the spinal dorsal horn of rats in each group. (E) RIP3 immunohistochemical staining in L4‐L6 spinal dorsal horns obtained in the different groups 14 days after surgery (magnification, 400×; Scale bar: 50 μm), (F) Quantification of Iba‐1 positive microglia cells. Values represent mean ± SD, *n* = 12 rats per group, *P < 0.05, **P < 0.01, ***P < 0.001 vs. Sham, ^#^ *P* < 0.05, ^##^ *P* < 0.01, ^###^ *P* < 0.001 vs. SNI. Student's *t*‐test and one‐way ANOVA were used for statistical analysis. All data were obtained from at least three independent experiments.

Studies have found that the occurrence and development of NP are not only limited to changes in neurons, but also closely related to the activation of glial cells [16]. To investigate whether the RIP3‐mediated necroptosis pathway is related to glial cells, immunofluorescence experiments were used to detect the distribution of astrocytes, microglia, and RIP3 protein in the SNI rat model. The results of immunofluorescence staining showed that RIP3 was coexpressed with the microglia marker Iba‐1, but not with the astrocyte marker GFAP in the spinal cord of rats in the SNI model group (Fig. 2A), and the number of microglia increased significantly in SNI rats, compared with the control group (Fig. 3E,F). Necroptosis mainly involves the receptor‐interacting protein kinase‐1 (RIP1), receptor‐interacting protein kinase 3 (RIP3), and mixed lineage kinase domain‐like protein (MLKL). Upon phosphorylation of RIP3 by RIP1, phosphorylated RIP3 activates MLKL, which eventually results in plasma membrane rupture and releases proinflammatory cellular contents to the extracellular space [17]. In order to further explore the role of RIP3‐mediated necroptosis pathway on microglia cells, interaction of RIP3 with RIP1, expression of RIP1, RIP3, and MLKL, and phosphorylation of RIP3 were measured by immunoblotting and immunoprecipitation assays on microglia cells extracted from the spinal cords of rats by flow cytometry. As shown in Fig. 2B, the interaction of RIP3 with RIP1, expression of RIP1, RIP3, and MLKL, and phosphorylation of RIP3 in the SNI group were increased significantly when compared with the sham group. These results indicate that the RIP3‐mediated necroptosis pathway promotes NP in rats via microglia in the SNI rat model.

### Intrathecal injection of the necroptosis pathway inhibitor Nec‐1 reduces neuropathic pain in SNI rats

To further study the relationship between necroptosis and NP in rats, the intrathecal injection technique was used to inject Nec‐1, a necroptosis inhibitor, into SNI rats on days 5–10 after the operation. The pain behavior results (PWMT) are shown in Fig. 3A. Compared with the SNI group, rats in the Nec‐1 group had a significantly higher PWMT 7, 10, and 14 days after the operation (*P* < 0.001). To explore whether Nec‐1 affects RIP3 expression, rat spinal dorsal horn tissues were obtained for western blot detection. The results showed that, compared with rats in the SNI group, the expression of RIP3 protein in the spinal dorsal horn of rats in the Nec‐1 group was significantly reduced (*P* < 0.05) (Fig. 3B,C). The ELISA test results showed that expression levels of TNF‐α, IL‐1β, and IL‐6 in the spinal dorsal horn of rats in the SNI group were significantly higher than those in the sham group (*P* < 0. 01). After injection of Nec‐1, expression levels of TNF‐α, IL‐1β, and IL‐6 in spinal dorsal horns were significantly decreased (*P* < 0.05) 4 days after the second administration of Nec‐1 (Fig. 3D). In addition, we further tested the effect of Nec‐1 on the regulation of microglia. As shown in Fig. 3E,F, Nec‐1 markedly reduced the expression of RIP3 and the number of microglia. These findings indicate that the necroptosis pathway inhibitor Nec‐1 effectively reduces NP in SNI rats by regulating microglia and reducing the level of proinflammatory factors.

## Discussion

The SNI model is a classical model for establishing NP in peripheral nerve injury [13]. In the present study, there was a significant decrease in mechanical stimulation threshold 3–5 days after surgery, confirming that SNI modeling was successful (Fig. 1A). With the passage of modeling time, the RIP3 protein increased significantly in the spinal cords of SNI rats (Fig. 1B–D), suggesting that necroptosis may play an important role in NP, but the molecular mechanism is unclear.

It has been established that the occurrence and development of NP are not limited to changes in neurons, but is also closely related to the activation of glial cells [16]. A large amount of evidence shows that glial cells can release and receive neurotransmitters through direct connections, synapses, and neuromodulation chemicals such as proinflammatory cytokines, chemokines, nitric oxide, prostaglandins, and excitatory amino acids [18, 19, 20, 21]. It is currently known that spinal microglia are activated following peripheral nerve or tissue damage [22]. Although proinflammatory cytokines and chemokines important for pathological pain were released upon induction of the pain model, the molecular mechanisms that regulate microglia activation and their interplay with necroptosis are not well‐known [23, 24]. Interestingly, we found that RIP3, the core regulatory protein of necroptosis, is differentially expressed in microglia, not in astrocytes, and the number of active microglia also increased in SNI models of NP (Fig. 3E,F). Also, the interaction of RIP3 with RIP1, expression of RIP1, RIP3, and MLKL, and phosphorylation of RIP3 in the microglia of SNI group were increased significantly when compared with the sham group (Fig. 2B). These results suggest that necroptosis promotes NP via microglia. At the same time, the mechanical pain response of rats in the experimental group was significantly enhanced 5 days after intrathecal injection of Nec‐1, suggesting that intrathecal injection of Nec‐1 can reduce the mechanical pain response caused by SNI in rats. Similarly, a recent study from Liang *et al*. [11] addressed the similar question using the chronic constriction injury pain model. They demonstrated that necroptosis was an important mechanism of cell death in NP induced by peripheral nerve injury. However, in their research, they did not clarify on which specific cells the necroptosis occurred.

As a key protein in necroptosis, RIP3 forms necrosomes with RIP1, which are thought to be molecular markers of necroptosis. Upon phosphorylation of RIK3 by RIP1, phosphorylated RIP3 activates MLKL, which eventually results in plasma membrane rupture and releases proinflammatory cellular contents to the extracellular space. Previous studies [25] have shown that necroptosis plays an important role in the process of early cell death following spinal cord injury, and the inhibition of necroptosis by Nec‐1 can reduce cell death in spinal cord tissue, protect neurons, and promote early recovery of motor function. Peripheral injury can cause activation of spinal dorsal horn glial cells and promote the release of proinflammatory factors, which play an important role in the generation and maintenance of NP. A large number of studies have shown that TNF‐α and IL‐1β play important roles in the generation and maintenance of NP. Gao *et al*. [26] found that PARP‐1‐regulated TNF‐α expression in the dorsal root ganglia and spinal dorsal horn contributes to the pathogenesis of NP in rats. Some studies report that RIP3 is a key protein regulating proinflammatory factors, which can specifically activate inflammasome 3 and promote caspase 1 activation, thus forming inflammasomes and leading to the secretion of inflammatory factors TNF‐α and IL‐1β [27]. To further investigate the role of IL‐1β, TNF‐α, and other inflammatory factors in the mechanism of NP, we used ELISA to detect the expression of IL‐1β, TNF‐α, and IL‐6 in rats. Our results show that IL‐1β, TNF‐α, and IL‐6 were all elevated in the SNI model group compared with the sham group, indicating that necroptosis may promote the level of TNF‐α, IL‐1β, and IL‐6 in the spinal cord. Intrathecal injection of Nec‐1 can significantly reduce levels of RIP3 (Fig. 3B,C), and the number of activated microglia in the spinal dorsal horn (Fig. 3E), as well as the expression levels of proinflammatory factors TNF‐α, IL‐1β, and IL‐6, suggesting that interplay between active microglia, necroptosis, and the inflammatory response are important factors in NP (Fig. 3D). However, there were some limitations in our study that still need to be thoroughly investigated. First, the molecular mechanism of the special activation of microglial RIP3 in NP still needs to be further elucidated. Also, further clarification is needed to determine whether the main source of proinflammatory cytokines comes from RIP3‐dependent necroptosis in microglia. In addition, the continuous activation time of RIP3 and whether the pain will not recur after administration of the Nec‐1 still need further research. Hence, understanding how the source of the necroptosis influences the response to NP still remains to be explored.

In summary, we found that RIP3 is upregulated in the nerve injury (SNI) rat model and that the RIP3‐mediated necroptosis pathway promotes NP in rats via microglia. Additionally, we demonstrated that intrathecal injection of Nec‐1 reduces NP in SNI rats by regulating microglia in the spinal cord and downregulating proinflammatory factors. Our research provides a theoretical basis for further research and provides new insights into the treatment of NP in the future.

## Conflict of interest

The authors declare no conflict of interest.

## Author contributions

JW conceived the study. PF searched the literature and collected the data. PF and GS performed the statistical analysis. PF drafted the manuscript. JW reviewed the manuscript. All authors have read and approved the final paper.

## Data Availability

All data generated or analyzed during this study are included in the article.
